# Metabolic Dysfunction-Associated Steatotic Liver Disease: Current Diagnostic Pathways, Noninvasive Fibrosis Assessment, and Therapeutic Strategies

**DOI:** 10.7759/cureus.108965

**Published:** 2026-05-16

**Authors:** Andrzej Bazan, Oliwer Plichta, Natalia Radzinska

**Affiliations:** 1 General Medicine, St. Anthony Hospital, Ząbkowice Śląskie, POL; 2 Medicine, Jan Mikulicz-Radecki University Hospital, Wroclaw, POL; 3 Midwifery, Faculty of Health Sciences, Wroclaw Medical University, Wroclaw, POL

**Keywords:** elastography, fibrosis, mash, masld, metabolic dysfunction-associated steatotic liver disease, noninvasive tests, obesity, type 2 diabetes

## Abstract

Metabolic dysfunction-associated steatotic liver disease (MASLD) is the current term for steatotic liver disease linked to cardiometabolic dysfunction. In clinical practice, the central task is not simply detecting steatosis but identifying patients with clinically important fibrosis who need closer assessment and treatment intensification. This narrative review summarizes current diagnostic pathways, sequential noninvasive fibrosis assessment, and therapeutic strategies for adults with suspected or confirmed MASLD, based on contemporary guidelines, consensus statements, and key studies. Current recommendations support targeted evaluation of adults with type 2 diabetes, obesity or excess adiposity with metabolic risk factors, unexplained liver enzyme abnormalities, or incidental steatosis. Initial assessment should define the metabolic context, quantify alcohol exposure, and exclude competing liver diseases. Fibrosis risk stratification relies on sequential noninvasive testing, usually beginning with simple blood-based scores and proceeding to elastography or other specialist tests in patients with indeterminate or higher-risk results. Treatment remains centered on weight reduction and cardiometabolic risk management, with additional roles for selected obesity- and diabetes-directed therapies, liver-directed pharmacotherapy in appropriate patients, and bariatric intervention in selected individuals. A fibrosis-focused, stepwise pathway shared across primary care, diabetes and obesity medicine, and hepatology is the most practical current model for MASLD care.

## Introduction and background

Metabolic dysfunction-associated steatotic liver disease (MASLD) is the current term for steatotic liver disease (SLD) associated with cardiometabolic dysfunction, and metabolic dysfunction-associated steatohepatitis (MASH) denotes its inflammatory form. The revised nomenclature places liver disease within the broader SLD framework and replaces older labels that were largely exclusion-based. Clinically, this matters because MASLD lies at the intersection of obesity, type 2 diabetes, dyslipidemia, and cardiovascular risk rather than within hepatology alone [1,2].

In routine care, the central problem is not merely the detection of steatosis. What changes the prognosis is the recognition of patients with clinically relevant fibrosis. Current guidance, therefore, supports targeted case finding and fibrosis risk stratification in adults with type 2 diabetes, obesity or excess adiposity with additional metabolic risk, unexplained liver enzyme abnormalities, or imaging-detected steatosis, followed by stepwise noninvasive fibrosis assessment [2].

Management now extends beyond recognition of fatty liver to staged risk assessment and treatment selection. Lifestyle intervention and sustained weight reduction remain the foundation of care, but current pathways also integrate cardiometabolic risk reduction, obesity- and diabetes-directed pharmacotherapy, bariatric intervention in selected patients, and, in appropriately staged adults with non-cirrhotic fibrotic MASH, liver-directed treatment within current guidance frameworks [2]. This review, therefore, examines how adults with suspected or confirmed MASLD should currently be assessed and managed in routine practice, with emphasis on disease definition, who to evaluate, the initial diagnostic workup, sequential fibrosis assessment, referral logic, and stage-appropriate treatment.

This article was prepared as a structured narrative review rather than a systematic review. Targeted PubMed searches and source verification were updated in March 2026, with supplementary checks of official society and publisher websites to confirm current guidance documents. Representative search terms included “MASLD”, “MASH”, “steatotic liver disease”, “fibrosis”, “noninvasive tests”, “FIB-4”, “elastography”, “type 2 diabetes”, “obesity”, “resmetirom”, “semaglutide”, and “bariatric surgery”. Priority was given to recent clinical practice guidelines, consensus statements, care pathways, high-quality review articles, and selected pivotal primary studies directly relevant to diagnosis, fibrosis assessment, and treatment. The search frame emphasized publications from 2023 to March 2026, while older sources were retained when foundational or necessary to interpret evidence originally published under NAFLD/NASH terminology. Articles were selected for discussion when they materially informed current diagnostic pathways, fibrosis risk stratification, or treatment positioning. Because the aim was focused clinical synthesis rather than exhaustive evidence capture, formal systematic review methods, study-level risk-of-bias assessment, and quantitative synthesis were not applied.

## Review

The reviewed literature supports a pathway-based model of care. In current guidance, the key clinical task is to identify adults at risk of clinically relevant fibrosis, stage liver disease with accessible sequential noninvasive tools, and link fibrosis risk to treatment intensity and referral. The resulting pathway is inherently multidisciplinary: it begins in primary care, diabetes, obesity, or cardiometabolic practice and escalates to hepatology when advanced fibrosis, cirrhosis, diagnostic uncertainty, or treatment eligibility becomes relevant.

Current disease definition and nomenclature

Current guidance uses SLD as the umbrella term for disorders characterized by excess hepatic fat. Within that framework, MASLD replaces NAFLD, and MASH replaces NASH. The change followed a multisociety Delphi process and was subsequently incorporated into major practice guidance. Its purpose was not merely linguistic. The earlier NAFLD/NASH terminology relied heavily on exclusion and included wording considered stigmatizing by many stakeholders, whereas the newer framework identifies the dominant cardiometabolic context more directly. The term “steatohepatitis” was retained because it remains clinically and pathophysiologically useful [1,2].

Operationally, MASLD requires evidence of hepatic steatosis together with at least one of five cardiometabolic risk factors. In adults, these include overweight or increased waist circumference, impaired glucose metabolism or type 2 diabetes, hypertension, hypertriglyceridemia, and low high-density lipoprotein cholesterol. MASH denotes the steatohepatitis form within MASLD. The broader SLD framework also includes alcohol-related liver disease, MetALD for patients who meet metabolic criteria for MASLD but also have alcohol exposure above that allowed for pure MASLD, and less common specific-etiology or cryptogenic forms of SLD. This structure is more useful in practice than the older NAFLD framework because it allows clinicians to describe metabolic dysfunction without assuming that other liver insults are absent [1,2].

The nomenclature, therefore, changes how the disease is framed at the bedside. A patient with steatosis, type 2 diabetes, and clinically relevant alcohol intake no longer sits awkwardly between mutually exclusive labels; the overlap category MetALD makes that phenotype explicit. The SLD framework also acknowledges that metabolic dysfunction may coexist with other chronic liver diseases, so the diagnostic workup still has to define alcohol exposure, competing causes of liver injury, and the metabolic setting rather than stop at a single label [1,3].

The terminology change also affects how older evidence should be read. Available analyses suggest that most patients previously classified as having NAFLD satisfy current MASLD criteria, so older NAFLD/NASH studies remain broadly informative for present-day practice. The correspondence is not exact, however, and caution is needed in patients with moderate alcohol exposure, mixed etiologies, or steatosis without metabolic risk factors. In this review, MASLD and MASH are therefore used as the default terms, while NAFLD and NASH are retained only when discussing historical studies, older guidance documents, or source papers that cannot be translated more precisely into the new framework [3-6].

Case finding and initial diagnostic workup

Current practice favors targeted case finding and fibrosis risk stratification in high-risk groups rather than indiscriminate population-wide screening. Across recent guidance, the aim is to identify adults most likely to harbor clinically important fibrosis, not simply to label every case of steatosis. The highest-yield groups include adults with type 2 diabetes, obesity or abdominal obesity with additional metabolic risk factors, persistent or otherwise unexplained liver enzyme abnormalities, and those in whom steatosis is detected incidentally on imaging. Diabetes-focused documents use more proactive language than hepatology guidance, particularly in people with type 2 diabetes or prediabetes, but the practical convergence is that evaluation should focus on groups with the greatest likelihood of advanced fibrotic disease [2,7-10].

Once a patient enters the pathway, the first consultation should determine whether the presentation is compatible with MASLD and whether the phenotype appears straightforward or mixed. The history should cover weight trajectory, type 2 diabetes or prediabetes, hypertension, dyslipidemia, current and recent medications, and family history of diabetes or cirrhosis. Alcohol history deserves separate and explicit attention. Quantity matters, but so do pattern and duration of intake, because metabolic dysfunction and alcohol-related liver injury often coexist rather than compete as mutually exclusive explanations. Physical examination remains useful for documenting central adiposity, body mass index, and waist circumference, blood pressure, and cutaneous markers of insulin resistance such as acanthosis nigricans when present, as well as signs suggesting advanced chronic liver disease, including hepatomegaly, splenomegaly, ascites, peripheral edema, spider angiomas, or palmar erythema [2,8].

A baseline laboratory assessment should do more than document aminotransferases. A practical first-line panel includes a hepatic profile and complete blood count with platelets, together with an assessment of glycemic status, lipids, and renal comorbidity. Additional tests depend on the biochemical pattern and the clinical context, but the initial laboratory strategy should already begin to separate metabolic fatty liver from more advanced or competing liver disease. Liver enzymes also need to be interpreted cautiously. Persistent or otherwise unexplained abnormalities remain important triggers for evaluation, but normal aminotransferases do not exclude clinically significant fibrosis and should not be used as reassurance on their own [2,8].

If steatosis has already been identified incidentally, that finding should trigger etiologic assessment and fibrosis triage rather than passive acknowledgment. If steatosis is still undocumented, imaging can support the diagnosis, but its limitations matter. Conventional ultrasound remains widely available and clinically useful for the initial detection of steatosis in routine practice, but current American Association for the Study of Liver Diseases (AASLD) imaging guidance does not support it as sufficient for disease characterization, fat quantification, or fibrosis staging because sensitivity is limited across the disease spectrum. In most adults, the decisive step after recognizing steatosis is therefore not repeated fat quantification but movement into a structured fibrosis risk pathway [2,11,12].

The workup must also define what else may be contributing. The newer SLD framework allows MASLD to coexist with other liver diseases, so the presence of metabolic risk factors does not end the diagnostic process. Viral hepatitis, autoimmune liver disease, iron overload syndromes, Wilson disease, alpha-1 antitrypsin deficiency, and drug-induced steatosis or steatohepatitis should be considered when liver chemistry abnormalities are disproportionate, the phenotype is atypical, or the overall clinical course does not fit the expected metabolic pattern. This is also the point at which MetALD becomes clinically relevant: alcohol exposure should be measured, not assumed away [1-3,8].

In practice, the initial diagnostic workup is complete only when four questions have been answered: Is steatosis likely or documented? Is the metabolic profile sufficient to support MASLD? Is alcohol or another liver disease contributing? And should the patient now proceed to formal fibrosis risk stratification? Current care pathways are built around that final question, because fibrosis stage rather than steatosis alone determines prognosis, follow-up intensity, and the threshold for specialist involvement [2,7,10].

Noninvasive fibrosis assessment and sequential pathways

In MASLD, noninvasive assessment is used primarily to identify fibrosis that changes prognosis, follow-up intensity, and treatment strategy. Current pathways are therefore built around fibrosis risk rather than repeated confirmation of steatosis alone. Across recent guidance, sequential testing is preferred over a single-test approach: simple blood-based scores are accessible and useful for ruling out advanced fibrosis, but their specificity is limited in the intermediate range and in groups such as older adults and people with type 2 diabetes [2,11-13].

In nonspecialist settings, the preferred entry test in most current pathways is the fibrosis-4 index (FIB-4). It is inexpensive, widely available, and calculated from age, AST, ALT, and platelet count. In adults aged 36-65 years, a FIB-4 value below 1.3 generally supports low risk, 1.3-2.67 defines an indeterminate zone that requires a second step, and values above 2.67 make advanced fibrosis more likely. In adults older than 65 years, the lower threshold is shifted upward to 2.0, whereas values above 2.67 still suggest a higher risk. FIB-4 is also less reliable in adults younger than 35 years, so an alternative fibrosis assessment is reasonable in that group. These thresholds are best understood as operational pathway cutoffs for triage rather than diagnostic absolutes, and they should be interpreted in the context of age, metabolic risk profile, and the overall clinical picture. Patients classified as low risk can usually remain in primary or diabetes care with periodic reassessment, although the interval is a pragmatic, guideline-linked interval rather than a universal rule [2,9,11].

When FIB-4 exceeds the low-risk threshold, second-line testing should usually be performed with vibration-controlled transient elastography (VCTE) or the Enhanced Liver Fibrosis (ELF) test. These are the preferred secondary tools in most current pathways because they refine fibrosis risk more effectively than repeating simple blood scores alone. Operational thresholds used in the European Association for the Study of the Liver (EASL) and several current US pathways generally classify VCTE <8 kPa or ELF <7.7 as low risk, VCTE 8-12 kPa or ELF 7.7-9.8 as intermediate risk, and VCTE >12 kPa or ELF >9.8 as high risk. Again, these values should be read as pathway cutoffs for risk stratification and referral rather than as universal stand-alone diagnostic boundaries. MRI-based tests, particularly magnetic resonance elastography, are best reserved for unresolved cases, technical limitations of elastography, or specialist-level clarification. The main sequential pathway is summarized in Table 1 [2,11-13].

**Table 1:** Sequential noninvasive fibrosis assessment pathway in adults at risk for MASLD This table summarizes the shared practical core of current EASL-, AASLD-, ADA-, and AGA-aligned fibrosis-triage pathways in adults at risk for MASLD. Simplified pathway: at-risk group → FIB-4 → VCTE/ELF → referral or follow-up. The thresholds shown are operational cut points intended for sequential assessment and referral, not stand-alone diagnostic boundaries. FIB-4 is less reliable in adults younger than 35 years, and test performance varies with age, type 2 diabetes, obesity, and clinical context. VCTE may be limited by central adiposity or technical failure, and modality-specific thresholds should not be transferred across platforms. Referral thresholds may also vary somewhat across specialty pathways and local access to elastography or ELF testing. ADA, American Diabetes Association; AASLD, American Association for the Study of Liver Diseases; AGA, American Gastroenterological Association; EASL, European Association for the Study of the Liver; ELF, Enhanced Liver Fibrosis test; FIB-4, fibrosis-4 index; MASLD, metabolic dysfunction-associated steatotic liver disease; MRE, magnetic resonance elastography; NIT, noninvasive test; SWE, shear-wave elastography; VCTE, vibration-controlled transient elastography Created by the authors as an original synthesis of the cited guidelines and review literature.

Step	Population/trigger	Preferred tool(s)	Working cutoffs/interpretation	Usual next action
1. Entry into the pathway	Type 2 diabetes; obesity or abdominal obesity with at least one additional cardiometabolic risk factor; persistently elevated liver enzymes; or incidentally detected steatosis	Clinical assessment + routine laboratory workup	Not a staging step	Proceed to first-line fibrosis triage
2. First-line fibrosis triage in nonspecialist care	Adults entering the pathway	FIB-4	Age ≤65 years: <1.3 low risk; 1.3-2.67 second-step testing; >2.67 likely advanced fibrosis. Age >65 years: <2.0 low risk; 2.0-2.67 second-step testing; >2.67 likely advanced fibrosis	Low risk: continue nonspecialist care with periodic reassessment. Above the low-risk threshold: proceed to second-line testing
3. Second-line risk stratification	FIB-4 above the low-risk threshold, or persistent clinical suspicion despite low-risk FIB-4	VCTE or ELF	VCTE: <8 kPa low risk; 8-12 kPa intermediate risk; >12 kPa high risk. ELF: <7.7 low risk; 7.7-9.8 intermediate risk; >9.8 high risk	Low risk: remain in nonspecialist care with repeat risk assessment. Intermediate/high risk: hepatology referral or specialist clarification
4. Specialist-level clarification	Indeterminate, discordant, or technically unreliable second-line results	MRE, SWE, or other specialist noninvasive tests	Use modality-specific validated thresholds	Refine fibrosis stage and decide whether biopsy or specialist management is needed
5. Referral to hepatology	High-risk NITs, suspected cirrhosis, unresolved diagnostic uncertainty, or possible competing/coexisting liver disease	-	Direct referral is reasonable with a clearly high FIB-4 (>2.67). Diabetes-focused guidance also supports referral when FIB-4 >1.3 is accompanied by VCTE ≥8.0 kPa or ELF ≥9.8	Specialist staging, etiologic clarification, cirrhosis pathway, or treatment-eligibility assessment
6. Liver biopsy: selective role	Not routine	Liver biopsy	No routine cutoff applies	Consider when NITs are indeterminate or discordant, cirrhosis is strongly suspected, aminotransferases remain elevated, or another/concomitant liver disease is possible

The indeterminate zone is where pathways are least uniform. Current guidance allows two pragmatic options for patients with FIB-4 values between 1.3 and 2.67: proceed directly to elastography, especially when the value approaches the upper range or the patient has a high-risk phenotype, or intensify lifestyle and cardiometabolic management and repeat FIB-4 after an interval, with elastography if the value remains elevated. This flexibility reflects both resource differences and the limitations of FIB-4 in diabetes, obesity, and older age. Discordance between noninvasive results and the clinical picture should not be ignored. Patients with borderline scores but persistent suspicion of advanced chronic liver disease, unexplained biochemical abnormalities, or technically unreliable measurements should move to specialist review rather than remain in a low-intensity pathway [2,11,12].

Referral logic is therefore stepwise rather than automatic. Direct hepatology referral is reasonable when FIB-4 is clearly high, when second-line testing suggests clinically relevant fibrosis or probable advanced disease, when cirrhosis is suspected clinically, or when another liver disease remains possible. Diabetes-focused pathways are often more proactive than hepatology-centered pathways, and the threshold for referral may therefore vary somewhat by specialty setting and local access to elastography or ELF testing. The 2025 ADA consensus is more explicit in diabetes care and states that individuals with FIB-4 >1.3 plus VCTE-derived liver stiffness ≥8.0 kPa or ELF ≥9.8 should be considered for referral to liver specialists. Comparative studies also show that pathway design materially affects specialty workload: different sequential algorithms may miss similar numbers of patients yet generate very different referral burdens [7,10,14,15].

Liver biopsy now has a selective rather than a routine role. In most cases, biopsy is not required for the clinical management of MASLD, although it remains necessary for the definite diagnosis of steatohepatitis and may help exclude alternative liver diseases. In practice, biopsy is best reserved for indeterminate or conflicting noninvasive findings, persistent unexplained aminotransferase elevation, suspected concomitant liver disease, or situations in which histology would materially alter management. For most adults, however, a structured sequence of FIB-4 followed by elastography or ELF is sufficient to guide monitoring, referral, and therapeutic planning [2,11-13].

Therapeutic strategies

Current MASLD treatment is staged according to fibrosis severity, metabolic phenotype, and care setting. Lifestyle measures and treatment of obesity, type 2 diabetes, dyslipidemia, and cardiovascular risk remain the basis of care for all patients. In contrast, MASH-directed pharmacotherapy is relevant mainly for selected adults with non-cirrhotic fibrotic disease. In major guidance, no class-wide MASH-directed pharmacotherapy is currently recommended for cirrhosis, so treatment at that stage remains largely supportive and specialist-led [2,7,16].

Lifestyle and Weight Reduction

Sustained weight loss remains the most consistent intervention. Current EASL guidance recommends dietary and behavioral weight loss for liver benefit and proposes targets of at least 5% to reduce liver fat, 7-10% to improve liver inflammation, and at least 10% to improve fibrosis. Diet quality also matters: a Mediterranean-style eating pattern, lower intake of ultra-processed food, saturated fat, sugar-sweetened beverages, and fructose, together with regular physical activity tailored to ability, are all advised. The same guidance recommends preferably more than 150 minutes/week of moderate-intensity or 75 minutes/week of vigorous-intensity activity, with minimization of sedentary time [2,16].

Alcohol counseling should be part of treatment rather than treated as a separate issue. Current guidance recommends permanent cessation of alcohol in advanced fibrosis or cirrhosis. Structured behavioral support is often needed because long-term adherence remains the main practical limitation of lifestyle therapy [2,16].

Metabolic Comorbidity as Part of Liver Care

MASLD should not be managed as an isolated liver disorder. Current guidance treats glycemic control, obesity treatment, lipid lowering, blood pressure control, and cardiovascular risk reduction as part of liver care because cardiovascular disease remains the dominant competing cause of morbidity and mortality. GLP-1 receptor agonists, SGLT2 inhibitors, metformin, and statins should be used for their standard indications, while recognizing that not all of these agents are established MASH-directed therapies. Their role in many patients with MASLD is therefore clear as part of metabolic and cardiovascular risk management, even when they are not specifically recommended as liver-directed treatment [2,7,16].

Diabetes- and Obesity-Directed Pharmacotherapy

An important qualification is that the 2024 European guidance preceded later semaglutide phase 3 data and subsequent practice updates. The field then moved quickly. In the ESSENCE phase 3 trial, once-weekly semaglutide 2.4 mg improved both primary histologic endpoints at 72 weeks in adults with MASH and stage F2-F3 fibrosis. Subsequent AASLD guidance updates positioned semaglutide as a treatment option for selected adults with non-cirrhotic fibrotic MASH while excluding cirrhosis from that setting. Semaglutide should, therefore, be described as a stage-specific option for selected non-cirrhotic patients in a defined clinical context, with final positioning dependent on current guidance and local regulatory status, rather than as a general treatment for MASLD across all stages [17,18].

Pioglitazone remains relevant, especially in patients with type 2 diabetes, because evidence supports its benefit on steatohepatitis and glycemic control. The main limitation is that the fibrosis benefit has been less consistent, and weight gain remains a practical drawback. SGLT2 inhibitors are attractive in diabetes-associated MASLD because of cardiovascular and renal benefits and modest reductions in liver fat, but current guidance still stops short of treating them as proven MASH-directed therapies. Their place is therefore best described as metabolically valuable and clinically relevant but not established as standard guideline-supported MASH-directed treatment [7].

In diabetes-associated MASLD, current diabetes guidance is more proactive than earlier hepatology guidance and argues that obesity pharmacotherapy should be considered alongside lifestyle treatment in people with overweight or obesity because lifestyle treatment alone often fails to maintain the magnitude of weight loss usually needed to reverse steatohepatitis and fibrosis [7].

Liver-Directed Pharmacotherapy

Resmetirom marked the first shift from supportive care alone to disease-specific pharmacologic treatment in selected adults with non-cirrhotic fibrotic MASH. The phase 3 MAESTRO-NASH trial showed superiority over placebo for both steatohepatitis resolution and improvement in fibrosis. Subsequent AASLD guidance updates positioned resmetirom for selected adults with non-cirrhotic fibrotic MASH within current practice. It should therefore be described as a treatment positioned for appropriately selected non-cirrhotic patients after fibrosis staging and specialist assessment, not as a general therapy for MASLD across all stages. In practice, its use also requires attention to contraindications or drug interactions, review of lipid and thyroid-related considerations where relevant, and longitudinal monitoring according to current guidance and local labeling. This expands treatment options, but it does not eliminate the need for careful patient selection and follow-up [19,20].

Tirzepatide has produced substantial phase 2 histologic results, but its place in routine liver-directed management remains evolving and still depends on longer-term and more confirmatory data. At present, it is better presented as an emerging therapeutic approach rather than as an established guideline-supported MASH-directed treatment [21].

Bariatric and Endoscopic Interventions

In adults with MASLD and obesity who meet standard obesity-surgery criteria, bariatric surgery should be considered rather than postponed until liver disease advances. Current guidance states that bariatric surgery can induce durable liver benefits and improve type 2 diabetes and cardiometabolic risk factors, and recent meta-analytic evidence also supports meaningful improvement in liver fat and histologic activity after bariatric intervention. In this context, bariatric surgery is best presented as a guideline-supported option for selected patients with obesity rather than as a universal liver-directed intervention. In compensated advanced chronic liver disease or compensated cirrhosis, surgery requires expert multidisciplinary assessment, especially for clinically significant portal hypertension. Endoscopic bariatric therapies remain of interest, but current guidance does not yet support presenting them as established MASH-directed treatments. The principal stage-based treatment strategy is summarized in Table 2 [2,16,22].

**Table 2:** Therapeutic strategies in MASLD according to disease stage and metabolic phenotype This table separates core guideline-supported care from treatments currently positioned for selected patients and from emerging or evolving therapeutic approaches. Availability, approved indications, and reimbursement may vary by jurisdiction, particularly for newer therapies. GLP-1, glucagon-like peptide-1; MASLD, metabolic dysfunction-associated steatotic liver disease; MASH, metabolic dysfunction-associated steatohepatitis; SGLT2, sodium-glucose cotransporter-2 Created by the authors as an original synthesis of the cited guidance documents and therapeutic literature.

Clinical context	Main therapeutic priorities	Interventions with current support	Current treatment position	Main caveats
All adults with MASLD	Improve liver injury, reduce cardiometabolic risk, prevent progression	Mediterranean-style diet or similar high-quality dietary pattern; limit ultra-processed foods and sugar-sweetened beverages; regular physical activity; alcohol counseling	Guideline-supported core care for all patients	In overweight/obesity, sustained weight loss is the main lever for liver benefit; in advanced fibrosis/cirrhosis, alcohol should be stopped completely
MASLD with overweight or obesity	Weight reduction sufficient to improve liver and metabolic outcomes	Behavioral weight-loss intervention; structured nutrition support; exercise; obesity pharmacotherapy when indicated	Guideline-supported foundational care	Weight-loss targets are practical rather than absolute; long-term adherence remains the main limitation
MASLD with type 2 diabetes and/or obesity, without cirrhosis	Treat metabolic disease as part of liver care	Metformin, GLP-1 receptor agonists, SGLT2 inhibitors, and statins according to standard indications; pioglitazone may be considered in selected non-cirrhotic MASH	Guideline-supported metabolic care, but not all agents are established for MASH-directed therapy	SGLT2 inhibitors and metformin should not be presented as proven MASH-directed treatment; pioglitazone is limited by weight gain and a less secure fibrosis signal
Non-cirrhotic fibrotic MASH (roughly F2-F3)/treatment-eligible disease	Add liver-directed therapy in appropriate patients while continuing lifestyle and metabolic care	Resmetirom in selected adults with non-cirrhotic fibrotic MASH; semaglutide in selected adults with non-cirrhotic fibrotic MASH where consistent with current guidance and local regulatory status	Guideline-positioned or label-consistent therapy for selected patients	Positioning depends on guidance framework, jurisdiction, and careful staging; contraindications, drug interactions, and monitoring requirements should be reviewed; combination sequencing remains unsettled
Emerging or evolving non-cirrhotic MASH approaches	Potential disease modification in selected patients	Tirzepatide and other rapidly evolving therapeutic approaches discussed in the current literature	Emerging/evolving; not established as standard guideline-supported MASH-directed therapy	Should not be presented as routine care where guidance, regulatory positioning, or reimbursement remain incomplete
MASLD with obesity meeting bariatric criteria	Durable weight loss, diabetes improvement, long-term cardiometabolic benefit	Bariatric surgery should be considered in non-cirrhotic MASLD; may be considered in selected compensated advanced disease after expert review	Guideline-supported in selected patients	Endoscopic bariatric procedures are not yet established as MASH-directed therapy; portal hypertension and liver stage must be assessed
Compensated advanced chronic liver disease/compensated cirrhosis	Prevent decompensation, preserve nutrition and muscle mass, treat comorbidity safely, start surveillance pathways	Adapt diet and exercise to sarcopenia risk; moderate weight reduction only when appropriate; metformin may be used with preserved renal function; GLP-1 receptor agonists may be used in Child-Pugh A according to indication; SGLT2 inhibitors may be used in Child-Pugh A-B; statins can be used	Specialist-led supportive management	No class-wide guideline-supported MASH-directed pharmacotherapy is currently recommended for cirrhosis in major guidance; treatment requires careful drug selection and surveillance planning
Decompensated cirrhosis/transplant pathway	Prevent complications, maintain nutrition, assess for transplantation	High-protein diet and late-evening snack when sarcopenia or decompensation is present; transplant-oriented hepatology care; alcohol abstinence	Supportive and transplant-focused care	Bariatric surgery is contraindicated outside transplant-context discussion; standard metabolic drugs may need restriction.

What Changes in Advanced Fibrosis or Cirrhosis

Once cirrhosis is established, the treatment goal broadens from reversing steatohepatitis to preventing decompensation and managing complications. No MASH-directed pharmacotherapy can currently be recommended for the cirrhotic stage as a class in major guidance. Nutritional advice should be adapted to disease severity and sarcopenia risk, with a high-protein diet and late-evening snack in sarcopenic or decompensated cirrhosis and only moderate weight reduction in compensated cirrhosis with obesity. Drug choice also changes: metformin can be used in compensated cirrhosis with preserved renal function, GLP-1 receptor agonists can be used in Child-Pugh A according to indication, SGLT2 inhibitors can be used in Child-Pugh A-B, and statins remain acceptable in compensated disease. Their use in this setting should be described as supportive metabolic management rather than as established cirrhosis-stage MASH-directed therapy [2,16,18,20].

Practical clinical decision-making

Current guidance indicates that MASLD should be managed through a shared pathway rather than through isolated specialty encounters. Most adults with MASLD are first seen in primary care, diabetes, endocrinology, obesity medicine, or cardiology clinics rather than in hepatology. Current pathway documents, therefore, frame MASLD as a condition that requires stepwise triage in non-hepatology settings, with selective escalation to specialist liver care when fibrosis risk, diagnostic uncertainty, or cirrhosis-related issues justify it [2,10].

In primary care, diabetology, and obesity medicine, the practical task is to identify at-risk adults, confirm the metabolic context, exclude obvious competing causes, and perform first-line fibrosis triage without delay. FIB-4 should ideally be generated automatically from routine laboratory data rather than being relied on only when a clinician remembers to calculate it. Patients in the low-risk range can usually remain in nonspecialist care, where the priority is sustained weight reduction, treatment of diabetes and obesity, cardiovascular risk reduction, and periodic reassessment. Diabetes-focused pathways are often more proactive than hepatology-centered pathways, and recommended follow-up intervals should therefore be understood as pragmatic, guidance-linked intervals rather than universal rules. In practice, guidance commonly suggests reassessment every one to two years in patients with type 2 diabetes, prediabetes, or at least two metabolic risk factors, and every two to three years in lower-risk individuals without diabetes and with fewer metabolic risk factors. Patients with indeterminate or high-risk results should move promptly to second-line assessment with elastography or ELF, depending on local access [9-11].

The hepatology pathway begins when noninvasive tests suggest clinically relevant fibrosis, when results are discordant, when aminotransferase abnormalities remain unexplained, or when another liver disease remains possible. At that stage, the goal is no longer only to confirm MASLD but to refine the stage, exclude mixed or alternative etiologies, decide whether biopsy is needed, and determine whether the patient has entered a cirrhosis pathway or may be eligible for treatment directed at non-cirrhotic fibrotic MASH [2,11,12].

Follow-up should also be risk-based rather than uniform. Guidance recommends assessment of related comorbidities and cardiovascular risk at diagnosis and at regular follow-up, with repeat laboratory and physical examination-based review rather than liver-focused follow-up alone. Serial noninvasive testing can be useful, but the same method, device, and laboratory platform should be used whenever possible because technical variability complicates interpretation of change over time [2,7,10].

A workable pathway, therefore, depends on multidisciplinary coordination. Liver disease, diabetes, obesity, dyslipidemia, hypertension, kidney disease, sleep apnea, and cardiovascular risk are tightly linked and cannot be managed well in parallel silos. Recent pathway documents were designed for use across primary care, diabetology/endocrinology, cardiology, obesity medicine, and hepatology. In practice, this means that nutrition support, obesity treatment, alcohol counseling, diabetes care, and liver-risk stratification should be connected within one pathway rather than delivered as separate referrals with no shared plan [2,7,10,23].

Implementation remains a major barrier, and this is where practical systems matter. In a pilot study of an electronic decision aid embedded in primary care, automated FIB-4 assessment and guideline-directed next-step recommendations improved adherence to recommended management while increasing hepatology referrals only modestly. The study was small and should not be overinterpreted, but it supports an important practical point: MASLD pathways work better when risk calculation and next-step recommendations are built into routine clinical workflow rather than left to memory [24].

Unresolved areas and evidence gaps

The diagnostic and therapeutic framework of MASLD has advanced quickly, but several clinically important uncertainties remain. The first is how aggressively high-risk groups should be evaluated. Major guidance documents are aligned in supporting targeted evaluation of adults at increased metabolic risk, but they differ in how explicitly they frame this as screening versus case finding and fibrosis risk stratification. This distinction is especially relevant in type 2 diabetes, where diabetes-focused recommendations are often more proactive than hepatology-centered pathways [7,25,26].

A second unresolved area is the performance of noninvasive tests in the populations in whom they are most often applied. The diagnostic performance of FIB-4 and liver stiffness measurement varies with age, type 2 diabetes, obesity, and clinical context, and currently used thresholds are best understood as operational pathway cutoffs rather than universal diagnostic boundaries. This remains an important limitation in everyday practice, particularly when treatment decisions are being made on the basis of sequential noninvasive assessment [27,28].

Therapeutic progress has created a different set of evidence gaps. Resmetirom and semaglutide have expanded treatment options for selected adults with non-cirrhotic fibrotic MASH, but important questions remain about sequencing, comparative effectiveness, combination use, and long-term monitoring. These uncertainties are compounded by differences in guidance language, regulatory positioning, and jurisdiction-dependent availability or reimbursement [18-20,26].

The treatment gap is greatest in advanced liver disease. Current guidance does not recommend class-wide MASH-directed pharmacotherapy for cirrhosis, leaving compensated advanced disease, portal hypertension, and decompensated cirrhosis largely within the domain of supportive care, surveillance, and transplant-oriented hepatology practice rather than proven disease-modifying treatment [2,18,20].

Implementation remains a final major barrier. Many patients and clinicians remain unaware of MASLD-related liver risk, lifestyle treatment is difficult to sustain in routine care, and real-world evidence still faces limitations in coding quality, confounding, and outcome capture. Future progress will therefore depend not only on new therapies but also on better harmonization of care pathways, more pragmatic implementation strategies, and outcome-focused studies in routine clinical populations [7,10,24,29,30].

Limitations

This review has several limitations. It was designed as a structured narrative review rather than a systematic review, so study selection was prioritized rather than exhaustive. The therapeutic field is also evolving quickly, particularly for newer MASH-directed agents, and treatment positioning may differ across guidance documents and jurisdictions. In addition, current recommendations are not fully harmonized across specialties, which affects terminology, referral thresholds, and treatment framing.

## Conclusions

MASLD should now be approached as a shared cardiometabolic and liver disorder rather than as an isolated hepatology diagnosis. In routine practice, the key task is not the detection of steatosis alone but the identification of patients with fibrosis that changes prognosis, follow-up intensity, and treatment strategy. A pathway that begins with targeted case finding in high-risk groups and proceeds through sequential noninvasive fibrosis assessment across primary care, diabetes and obesity medicine, and hepatology is the most practical current model of care. Treatment should be staged in the same way. Lifestyle-based weight reduction and management of obesity, type 2 diabetes, dyslipidemia, and cardiovascular risk remain the foundation of care for all patients. At the same time, treatment options for selected adults with non-cirrhotic fibrotic MASH have expanded, and current guidance increasingly integrates stage-specific liver-directed pharmacotherapy and selected obesity- and diabetes-directed therapies within defined clinical settings. Once cirrhosis is established, however, management shifts toward specialist hepatology care, complication prevention, and surveillance because disease-modifying options remain limited.

Important uncertainties remain. Current guidance is not fully harmonized on how intensively high-risk groups should be evaluated; the performance of noninvasive tests varies with age, type 2 diabetes, and obesity, and the long-term sequencing or combination of newer therapies remains unsettled. These gaps strengthen the need for clearer pathways, better implementation, and outcome-focused research.
